# Maternal microbiota communicates with the fetus through microbiota-derived extracellular vesicles

**DOI:** 10.1186/s40168-023-01694-9

**Published:** 2023-11-13

**Authors:** Anna Kaisanlahti, Jenni Turunen, Nadiya Byts, Anatoliy Samoylenko, Genevieve Bart, Nikke Virtanen, Mysore V. Tejesvi, Artem Zhyvolozhnyi, Sonia Sarfraz, Sohvi Kumpula, Jenni Hekkala, Sonja Salmi, Olga Will, Johanna Korvala, Niko Paalanne, Pande Putu Erawijantari, Marko Suokas, Tuula Peñate Medina, Seppo Vainio, Oula Peñate Medina, Leo Lahti, Terhi Tapiainen, Justus Reunanen

**Affiliations:** 1 Biocenter Oulu, University of Oulu, 90220 Oulu, Finland; 2 Research Unit of Translational Medicine, University of Oulu, 90220 Oulu, Finland; 3 Research Unit of Clinical Medicine, University of Oulu, 90220 Oulu, Finland; 4 Laboratory of Developmental Biology, Disease Networks Research Unit, Faculty of Biochemistry and Molecular Medicine, University of Oulu, 90220 Oulu, Finland; 5 Ecology and Genetics, Faculty of Science, University of Oulu, 90570 Oulu, Finland; 6 Section Biomedical Imaging, Department of Radiology and Neuroradiology, University Hospital Schleswig-Holstein Campus Kiel, Kiel University, 24105 Kiel, Germany; 7 Department of Pediatrics and Adolescent Medicine, Oulu University Hospital, 90220 Oulu, Finland; 8 Department of Computing, University of Turku, 20014 Turku, Finland; 9 Institute for Experimental Cancer Research, Kiel University, 24105 Kiel, Germany; 10 Kvantum Institute, University of Oulu, 90570 Oulu, Finland; 11 Lonza Netherlands B.V., 6167 RB Geleen, Netherlands

**Keywords:** Gut microbiota, Extracellular vesicles, Fetal environment, Amniotic fluid, Fetal microbiota, Intestine

## Abstract

**Background:**

Reports regarding the presence of bacteria in the fetal environment remain limited and controversial. Recently, extracellular vesicles secreted by the human gut microbiota have emerged as a novel mechanism for host-microbiota interaction. We aimed to investigate the presence of bacterial extracellular vesicles in the fetal environment during healthy pregnancies and determine whether extracellular vesicles derived from the gut microbiota can cross biological barriers to reach the fetus.

**Results:**

Bacterial extracellular vesicles were detectable in the amniotic fluid of healthy pregnant women, exhibiting similarities to extracellular vesicles found in the maternal gut microbiota. In pregnant mice, extracellular vesicles derived from human maternal gut microbiota were found to reach the intra-amniotic space.

**Conclusions:**

Our findings reveal maternal microbiota-derived extracellular vesicles as an interaction mechanism between the maternal microbiota and fetus, potentially playing a pivotal role in priming the prenatal immune system for gut colonization after birth.

Video Abstract

**Supplementary Information:**

The online version contains supplementary material available at 10.1186/s40168-023-01694-9.

## Background

The human gut microbiota comprises a diverse ecosystem with thousands of microbes, each with diverse functional activities. Its significance in human health is a growing field in biomedical research. One recent object of keen interest in the field of host-microbiota communication has been the extracellular vesicles (EVs) secreted by the gut microbiota [1–8]. EVs, membrane-enclosed particles secreted by almost all cell types, can transport various biomolecules across biological barriers [9].

The concept of the fetal microbiome remains a topic of debate. Although previous studies have reported the presence of bacterial DNA in the placenta and amniotic fluid [10–12], the idea of a distinct fetal microbiota, comprising whole-cell bacteria, has been met with skepticism [13–19]. The effects of EVs derived from single laboratory-cultured pathogens at the feto-maternal interface [20, 21] and the modulation of bacterial EV secretion in urine during pregnancy have already been shown in humans [22]. However, data on microbial EVs in healthy pregnancies are scarce [23–25], and microbiota-derived EVs have not been investigated in the fetal environment.

We hypothesized that maternal microbiota-derived EVs cross biological barriers and reach the fetus. We aimed to characterize bacterial EVs in the gut and amniotic fluids of healthy pregnant women. Additionally, we explored whether maternal gut microbiota-derived EVs interact with the fetus in a mouse model.

## Methods

### Collection of amniotic fluid and fecal samples from pregnant women

Amniotic fluid (*n* = 26) and fecal samples (*n* = 25) were obtained from 28 pregnant women undergoing elective cesarean section delivery after a term pregnancy at Oulu University Hospital, Oulu, Finland. The clinical characteristics of the pregnant women are presented in Additional file 1: Table S1. The sampling procedure has been described in detail previously [13]. In brief, amniotic fluid samples were collected under sterile conditions during the Caesarean section by the obstetrician, while fecal samples were obtained from the mothers before the Caesarean section by a nurse. Both the amniotic fluid and fecal samples were immediately stored at − 20 °C upon collection. Only women who provided written informed consent for these procedures were enrolled. The research protocol received approval from the Ethical Committee of the Northern Ostrobothnia Hospital District at Oulu University Hospital, Finland (decision number EETTMK: 3/2016). All clinical aspects of the study were conducted in accordance with relevant guidelines and regulations.

### Isolation of EVs from amniotic fluid samples

EVs were isolated from amniotic fluid samples by ultracentrifugation and subsequent density gradient ultracentrifugation, as previously described [4, 26]. Briefly, 10 mL of each sample was purified from solid material by centrifugation at 10,000 × *g* for 30 min at 4 °C. The samples were then filtered through a 0.8-μm syringe filter (Minisart®). The EVs were isolated by ultracentrifugation at 100,000 × *g* for 18.5 h using an Optima L-100/L90 ultracentrifuge (Beckmann) equipped with a SW 41 Ti swinging bucket rotor (12 mL; Beckmann). The resulting pellet containing EVs was dissolved in phosphate-buffered saline (PBS) and further enriched for bacterial EVs through OptiPrep™ gradient density ultracentrifugation using fractions 6 and 7, which were washed with PBS and used for downstream analysis [4, 26, 27]. The isolated EVs were stored at – 20 °C until analysis. Fractions 6 and 7, obtained from the gradient overlaid with PBS, were used as a negative control in the proteomics analysis.

### Isolation of EVs from maternal fecal samples

EVs were isolated from the maternal fecal samples through a series of filtration, size-exclusion chromatography, and density gradient ultracentrifugation steps, as previously described [4, 26, 27]. Briefly, 4 g of fecal material from each maternal sample was suspended in PBS and subjected to repeated centrifugation at 14,000 × *g* for 30 min at 4 °C to remove solid material. Subsequently, the samples were filtered through a 40-μm nylon filter (Falcon) and a 0.45-μm PES filter (Biofil) and concentrated using Centricon® Plus 70 filter devices (Millipore). For size-based removal of feces-specific contaminants, such as lipoproteins and chylomicrons, EVs were isolated from the concentrated samples through size-exclusion chromatography using Exo-Spin™ Mini-Columns (Cell Guidance Systems). The preparations were further enriched for bacterial EVs using OptiPrep™ gradient density ultracentrifugation on fractions 6 and 7, previously washed with PBS, and these fractions were used for downstream analysis [4, 26, 27]. The isolated fecal EVs were stored at − 20 °C until analysis. Fractions 6 and 7, obtained from the gradient overlaid with PBS, were used as a negative control in the proteomic analysis.

### Transmission electron microscopy (TEM) analysis of isolated EVs

Transmission electron microscopy (TEM) imaging was conducted as previously described [27]. EVs isolated from the amniotic fluid and fecal samples were deposited on Formvar carbon-coated and glow-discharged copper grids. The samples were fixed to the grids using 1% glutaraldehyde and negatively stained with a 2% methylcellulose–0.4% uranyl acetate solution. The sample grids were then examined and images of them were captured with Tecnai G2 Spirit 120 kV TEM, coupled with Veleta and Quemesa CCD cameras (Tissue Imaging Center, Biocenter Oulu, Oulu, Finland).

### Nanoparticle tracking analysis (NTA) of EVs

The concentrations and size distributions of the isolated EVs were characterized through nanoparticle tracking analysis (NTA) using a NanoSight NM300 instrument (Malvern Panalytical) as previously described [27]. The instrument was equipped with a 405-nm laser, a syringe pump, and NTA software, version 3.4.4 (Malvern Panalytical). For optimal measurements, the samples were pre-diluted with PBS. The obtained EV sizes were presented graphically, along with their mean (SD) and mode (SD), and the size distributions of the EVs were represented as the means (SD) of the D10, D50, and D90 values. The sizes and size distributions of the amniotic fluid EVs and maternal fecal EVs were then compared using a paired two-tailed *t*-test. Figures were created and statistically analyzed using GraphPad Prism (version 9.3.1).

### DNA extraction from amniotic fluid and feces

Total DNA was extracted from the fecal and amniotic fluid samples using the DNeasy PowerSoil Pro kit (Qiagen). The fecal samples were homogenized with stainless steel beads in a Tissuelyzer. Approximately 200 mg of fecal sample was dissolved in 1 mL of PBS in a 2-mL centrifuge tube. The sample was shaken at 25 Hz for 2 min, incubated on ice for 2 min, and shaken once again for 1 min at 25 Hz. The homogenate was further processed following the manufacturer’s protocol. For amniotic fluid, a 2.5-mL cryotube was centrifuged at 8609 × *g* for 20 min, and the supernatant was discarded. The resulting pellet was horizontally homogenized according to the manufacturer’s protocol following which the same protocol was continued for the rest of the DNA extraction process. The fecal samples were eluted in 100 μL of the elution reagent, and the amniotic fluid samples were eluted in 50 μL. A total of 5 negative controls (sterile water, HyClone™ HyPure, Thermo Scientific) were processed alongside these samples.

### Extraction of total RNA from EVs, and cDNA synthesis

We chose to characterize microbiota-derived EVs through RNA analysis, specifically using 16S ribosomal ribonucleic acid (rRNA) gene sequencing. This choice stemmed from the limited and inconsistent data regarding the presence of genomic DNA in EVs [28, 29]. Total RNA extraction was performed on both amniotic fluid and fecal EV samples using the exoRNeasy Serum Plasma Midi Kit (Qiagen) following the manufacturer’s instructions. Subsequently, cDNA synthesis was conducted using 20 ng of RNA using the iSCRIPT™ cDNA synthesis kit (Bio-Rad) according to the manufacturer’s protocol. Additionally, we included the S-D-Bact-0341-b-S-17 primer (5′-CCTACGGGNGGCWGCAG-3′) specific to the universal 16S rRNA gene in the reaction mix [30].

### Polymerase chain reaction (PCR) and 16S rRNA gene sequencing

After RNA analysis, we conducted PCR and 16S rRNA gene sequencing on the EVs isolated from the maternal amniotic fluid and fecal samples. The target region was the V4–V5 hypervariable segment of the 16S rRNA gene, and we used primer pairs 519F (5′-CAGCMGCCCGCGGTAATWC-3′) and 926R (5′-CCGTCAATTCCTTTRAGTTT-3′) [31, 32]. The 519F primer was individually barcoded for each sample to facilitate pooling during sequencing. For the PCR reactions, we used the Phusion Flash High-Fidelity PCR master mix (Thermo Scientific) and followed the manufacturer’s protocol for 50 μL reactions. Additionally, we included two negative controls (sterile water, HyClone™ HyPure, Thermo Scientific) and two mock community controls consisting of HM-782D, and Microbial Mock Community B (BEI resources, USA) in the PCR and sequencing run.

The PCR reactions were conducted using the Applied Biosystems™ Veriti 96-Well Thermal Cycler (Thermo Scientific). The cycle began with an initialization step of 3 min at 98 °C, followed by 30 cycles of reaction at an annealing temperature of 56 °C, and a final elongation step of 5 min at 72 °C. The PCR products were verified via agarose gel electrophoresis and imaging using a VersaDoc imaging machine from Bio-Rad. Subsequently, the PCR products were pooled, and purification was performed using AMPure XP (Beckman Coulter, CA, USA). The pool underwent electrophoresis in 1% agarose gel, from which the product was excised and purified using the MinElute Gel Extraction Kit (Qiagen). A second PCR was performed on the pool using the Phusion Flash High-Fidelity PCR master mix with 1 μM primers A and trP1. This second PCR comprised 7 cycles at an annealing temperature of 63 °C, with a final elongation step lasting 5 min. The PCR product was once again purified using AMPure XP, analyzed using Bioanalyzer, and the pool’s concentration was measured using a Quant-iT PicoGreen dsDNA Assay Kit (Thermo Fisher Scientific). Sequencing was performed using the IonTorrent PGM (Thermo Fisher Scientific).

### 16S rRNA gene-sequence analysis

The sequencing results were analyzed using QIIME2 (version 2021.2) [33]. To ensure data quality, reads shorter than 200 bp were excluded. The remaining reads were subjected to denoising and demultiplexing using DADA2, involving trimming at 15 and truncating at 250. Chimeric reads were rigorously filtered out [34]. Contaminant reads were removed using the R *decontam* package (version. 1.8.0) with reference to negative controls generated during DNA extraction and PCR. The prevalence-based method with a threshold of 0.5 was used for this purpose [35]. Taxa identified as *Cyanobacteria*, *Mitochondria*, *Eukaryota*, or *Archaea* were eliminated from the analysis. Additionally, known skin contaminants, *Corynebacterium* and *Cutibacterium*, were excluded. The mean read frequency was 9108, with a median frequency of 3914. The read data were rarefied, setting a sampling depth of 1609 based on the lowest read count after eliminating samples with insufficient read counts.

Within-sample diversity, referred to as alpha diversity, was assessed using the Shannon index and the count of observed features. The statistical significance of alpha diversity differences between the groups was evaluated using the Kruskal–Wallis *H* test, with *p* < 0.05 as the significance threshold. Pairwise differences between groups were determined via the pairwise Wilcoxon rank sum test, applying the Bonferroni correction within RStudio (version 2022.07.1 [36], R version 4.2.1 [37]). Additionally, community similarity, known as beta diversity, was visually represented through principal coordinate analysis based on Bray–Curtis dissimilarity. The significance of the group-wise differences in beta diversity was assessed using PERMANOVA. Relative abundances of taxa at the phylum and genus levels were computed using the SILVA database (version 138), presenting microbial taxa names as they appear in the database [38]. Differentially abundant taxa were identified using the analysis of composition of microbiomes (ANCOM) method [39]. The figures were drawn using RStudio (version 2022.07.1 [36], R version 4.2.1 [37]) with the R package ggplot2 (version 3.3.6) and GridExtra (version 2.3).

### Isolation of proteins from the EVs

Proteins from the EVs were isolated through methanol precipitation following previously described protocols [40]. In brief, the EV proteins were precipitated in a solution containing four times the sample volume of methanol, one time the sample volume of chloroform, and three times the sample volume of dH2O, achieved through centrifugation at 14,000 × *g* for 1 min. After removing the water and methanol mixture, the precipitates were washed with methanol and centrifuged at 20,000 × *g* for 5 min. The protein pellets were then air-dried horizontally at room temperature for 10–20 min and subsequently resuspended in 1 × Laemmli buffer. The resuspended proteins were boiled at + 95 °C for 5 min and loaded into a 12% SDS-PAGE gel. Electrophoresis was conducted at 110 V for 10–15 min, followed by fixation of the gel for 30 min in a solution containing 50% ethanol and 10% acetic acid. The fixed gel was stained overnight in a 1 × Sypro Ruby protein solution, protected from light, and destained the following day in 5% acetic acid for 5 min. Finally, the gel was immersed in dH2O for 15 min, and the protein bands were excised under UV light and stored in 20 μL of dH2O in Eppendorf tubes.

### Mass spectrometry analysis of EV proteins

The analysis of EV proteins via mass spectrometry was performed at the Turku Proteomics Facility, Turku, Finland. Peptides that had been digested with trypsin were re-suspended in 15 μL of formic acid, and 5 μL of this solution was subjected to analysis. Liquid chromatography-electrospray ionization-tandem mass spectrometry analyses were conducted using a nanoflow HPLC system (Easy-nLC1200, Thermo Fisher Scientific) coupled with a Q Exactive HF mass spectrometer (Thermo Fisher Scientific, Bremen, Germany) equipped with a nano-electrospray ionization source. Peptides were initially loaded onto a trapping column and subsequently separated on a 15-cm C18 column (75 μm × 15 cm, ReproSil-Pur 5 μm 200 Å C18-AQ, Dr. Maisch HPLC GmbH, Ammerbuch-Entringen, Germany). The mobile phase comprised water with 0.1% formic acid (solvent A) and acetonitrile/water (80:20, v/v) with 0.1% formic acid (solvent B). Peptides were eluted with a linear 20-min gradient of solvent B, ranging from 8 to 43%. Mass spectrometry data were acquired automatically using Thermo Xcalibur 4.1 software (Thermo Fisher Scientific). The information-dependent acquisition method comprised an Orbitrap mass spectrometry survey scan, covering a mass range of 300–2000 m/z, followed by high-energy collisional dissociation fragmentation of the 10 most intense peptide ions.

Proteomic data analysis was conducted using Peaks Studio software (version 10.6). Searches were performed against the UniProt Swissprot and UniProt trEMBL databases (UniProt release 2021_04), with a parent mass error tolerance set at 10.0 ppm and a fragment mass error tolerance at 0.02 Da [41]. False discovery rates for both peptide and protein identifications were set at 1.0%, and only the top proteins in each group were considered. A protein was considered identified if it was represented by at least one unique peptide, and the total protein coverage from supporting peptides was ≥ 1%. Negative controls were analyzed in parallel with EV-derived proteins, and any proteins identified in the control samples were excluded from the EV protein identification results. Taxonomy in the analysis results was referenced according to the names in UniProt release 2021_04. Data from the identification results were processed in R using the script presented in Additional file 2, accompanied by metadata in Additional file 3, and a description of the script in Additional file 4. Figures were drawn using RStudio (version 2022.02.3 [36], R version 4.2.0 [37]) with the ggplot2 package (version 3.3.6).

### Biodistribution analysis of human fecal EVs in pregnant mice

EVs obtained from fecal samples of four pregnant women were labeled with Bodipy Texas red-ceramide (Invitrogen, Germany) [42, 43], following previously established protocols [44]. Briefly, 1 mM of dye (in dimethyl sulfoxide) was combined with EVs (5 μL of dye per 100 μL of EVs) and incubated for 30 min at 37 °C. The excess, unincorporated dye was removed from the labeled EVs through centrifugation of the mixture for 20 min at 27,000 × *g*, and the stained vesicles were reconstituted in PBS.

A 12-week-old female FVB/NRj mice (Source: Janvier Labs) were maintained under controlled temperature and humidity conditions, with a 12-h light–dark cycle and free access to food and water. This experiment received approval from the local animal experimentation ethics committee (MELUR), with license number (V 242–68909/2015 (87–6/15)), and adhered to the guidelines for animal care recommended by the University of Kiel, Germany.

For this study, four mice were used at the end of their first trimester of pregnancy. Fecal-derived EVs, labeled with Texas red, were isolated from pregnant women (100 μL per mouse, equivalent to approximately 10^8^ EVs per mouse) and administered to the mice through tail vein injection. Following 24 h of circulation, the animals were humanely euthanized via intraperitoneal injection [125 μL of a cocktail consisting of 0.6% ketamine (AVECO Pharmaceuticals, Boston, MA, USA) and 0.4% medetomidine (Pfizer Deutschland, Berlin, Germany) in 4.0% NaCl]. Subsequently, the mice were sacrificed, and their organs and fetuses were collected for ex vivo imaging using the NightOWL 983 system from Berthold Technologies (Bad Wildbad, Germany). The imaging process used excitation/emission filters of 740/790 nm for Texas Red, and images were generated. Regions of interest were defined around the organs, and the maximum relative fluorescence intensity voxel was measured and analyzed with the Indigo software. Muscle tissue served as a control for nonspecific targeting.

Statistical analysis of fluorescence intensity was conducted using GraphPad Prism (version 9.3.1) through the Kruskal–Wallis nonparametric test. The intensities of various organs and fetuses were visually represented in terms of means and standard deviations generated by GraphPad Prism (version 9.3.1).

### Statistical analysis

For comparing Nanosight results regarding amniotic fluid and fecal EV sizes and size distribution parameters, a paired, two-tailed *t*-test was applied using GraphPad Prism (version 9.3.1 In the analysis of 16S rRNA gene sequences, multigroup comparisons were performed using the Kruskal–Wallis test within QIIME2 (version 2021.2) [33]. Pairwise tests employed the Wilcoxon rank sum test with Bonferroni correction for multiple comparisons implemented in RStudio (version 2022.07.1 [36], R version 4.2.1 [37]). The significance of the group-wise differences in beta diversity was assessed through PERMANOVA, and differentially abundant taxa were identified using the analysis of the composition of microbiomes (ANCOM) method [39], both implemented in QIIME2 (version 2021.2) [33].

In the biodistribution assay, the Kruskal–Wallis test was used for multi-group comparisons to analyze fluorescence intensities of the fetus and maternal organs using GraphPad Prism (version 9.3.1).

## Results

### Maternal feces-derived EVs showed wider size distribution than amniotic fluid-derived EVs

**Fig. 1:**
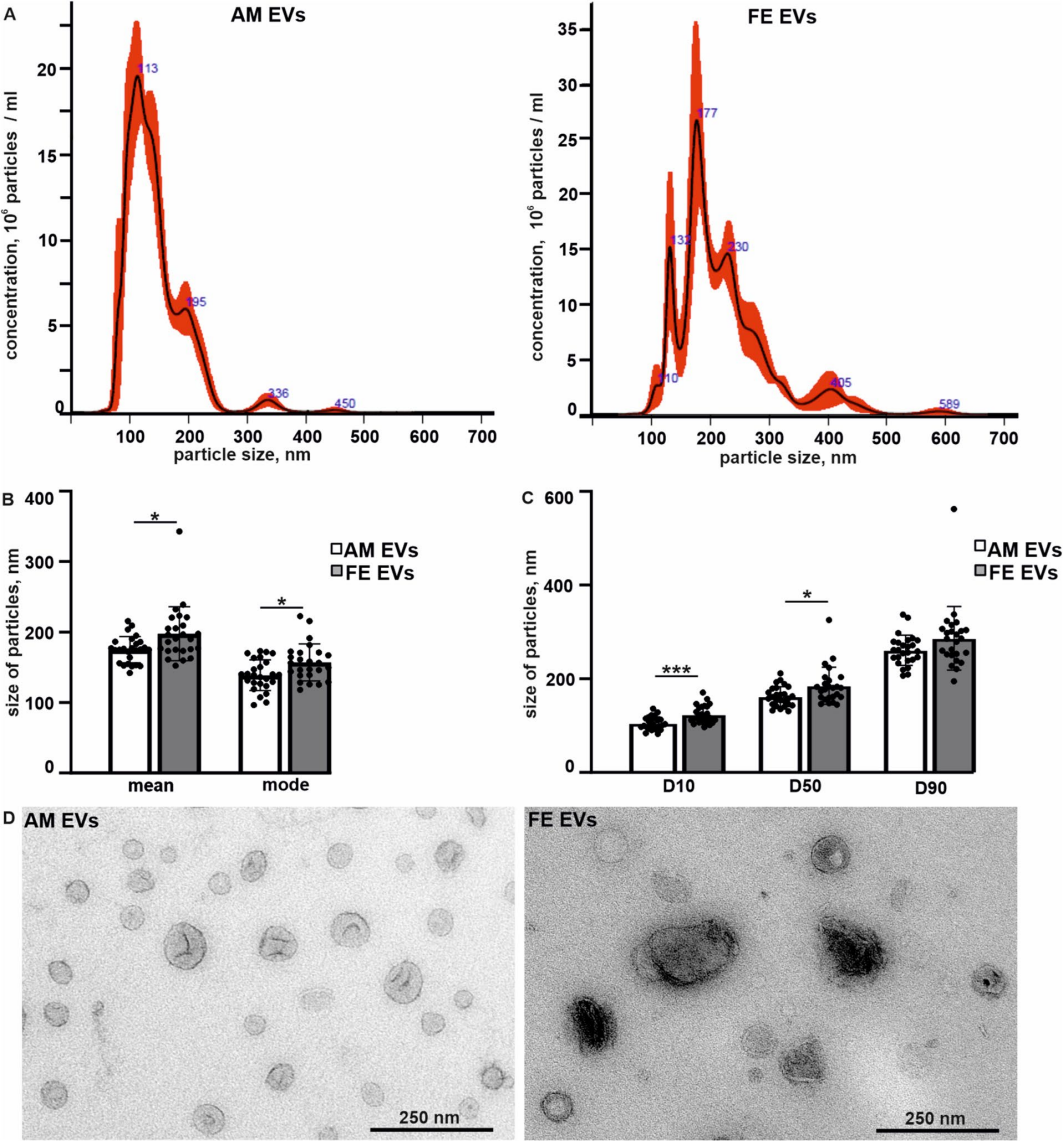
Extracellular vesicles of maternal fecal samples (FE EVs) show a wider size distribution as compared to amniotic fluid-derived extracellular vesicles (AM EVs). Representative size distribution of EVs derived from the amniotic fluid and feces in nanoparticle tracking analysis (NTA) (**A**). Analysis of the EV size as represented by the mean and mode, with error bars indicating the standard deviation, *n* = 26 amniotic fluid and *n* = 25 fecal samples, each dot representing one sample, *p < 0.05 and ***p < 0.001 in a paired, two-tailed *t*-test (**B**). The size distribution of EVs represented as the mean of the diameter 10th percentile (D10), diameter 50th percentile (D50), and diameter 90th percentile (D90) values corresponding to different percentages of the EV population under the given diameter, with error bars indicating standard deviation, *n* = 26 amniotic fluid and *n* = 25 fecal samples, *p < 0.05 and ***p < 0.001 in the paired, two-tailed *t*-test (**C**). Representative transmission electron micrographs of the amniotic fluid and fecal EVs (**D**)

The nanoparticle tracking analysis was conducted to characterize the EVs present in the amniotic fluid and feces of pregnant women. The analysis revealed concentrations of 10^8–9^ nanoparticles/mL in fecal extracts and 10^8–10^ nanoparticles/mL in amniotic fluids. Both sample types exhibited a substantial presence of particles smaller than 200 nm, consistent with the previously reported size range of small EVs. The mean particle sizes in the fecal EV samples were significantly larger than those in the amniotic fluid EV samples. Furthermore, the size distribution of particles in fecal EV samples was wider than that in amniotic fluid EV samples, with mean sizes ranging from 142 to 216 nm for amniotic fluid EVs and from 152 to 343 nm for fecal EVs (Fig. 1A–C). The EV size distributions within individual samples are presented in Additional file 1: Figure S2, S3). Transmission electron microscopy revealed bilayered membrane nanostructures typical of EVs in the fecal samples, and amniotic fluid EVs exhibited the “deflated balloon” phenotype (Fig. 1D) [45, 46].

### Protein cargo of bacterial EVs in amniotic fluid and maternal feces showed similarities in origin and functional characteristics

In total, 11,280 distinct peptides were identified from the UniProtKB swissProt database and 14,406 from UniProtKB trEMBL. This resulted in the identification of 3526 proteins in the amniotic fluid samples and 8417 proteins in the fecal EV samples. The protein identification results are available in Additional files 5 and 6 for UniProtKB SwissProt and UniProtKB TrEMBL, respectively. Specifically, 340 bacterial proteins and 3186 human proteins were identified in the amniotic fluid EV samples, whereas the fecal samples contained 7558 bacterial and 859 human protein identifications. On average, the amniotic fluid samples had 30 bacterial and 3186 human proteins per sample, whereas the fecal samples contained an average of 1333 bacterial and 60 human protein identifications. Statistics on protein identifications in both sample groups are presented in Additional file 1: Table S2.

**Fig. 2:**
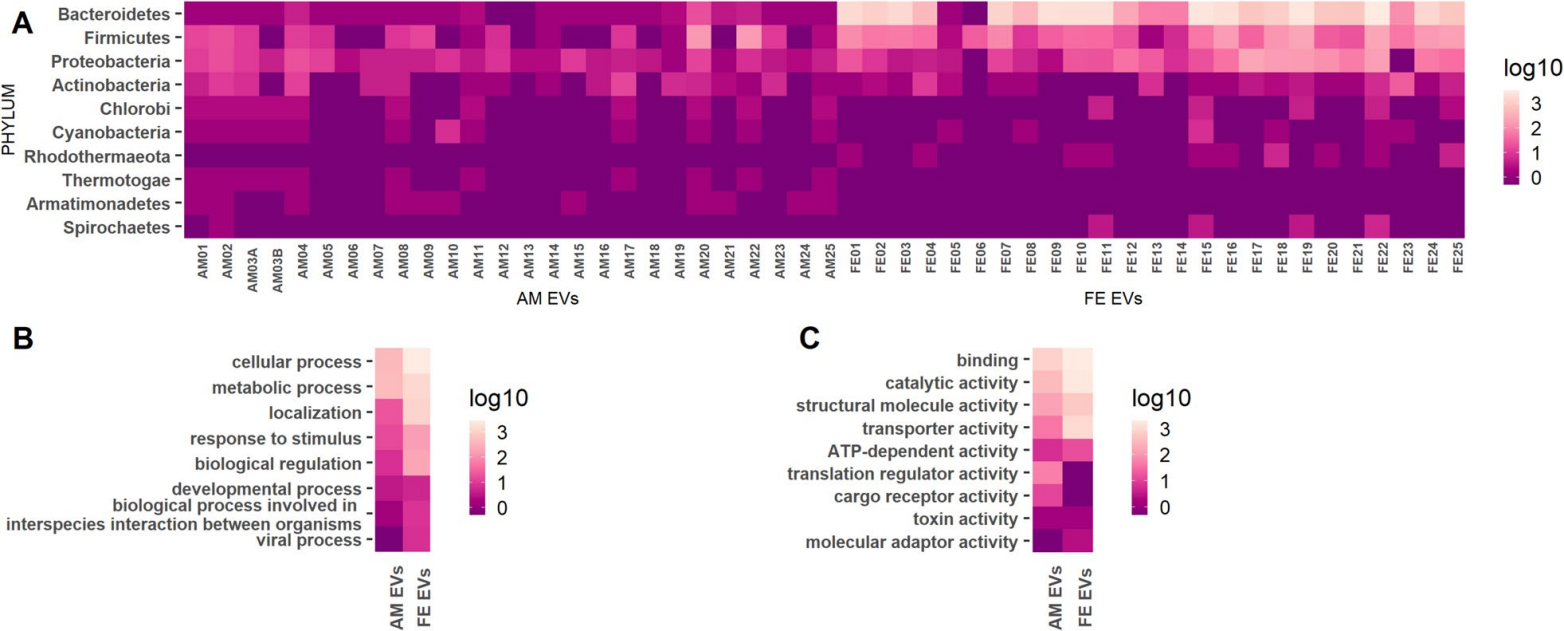
Bacterial proteins in amniotic fluid extracellular vesicles (AM EVs) and maternal fecal extracellular vesicles (FE EVs) share taxonomic and functional characteristics. Taxonomy of the bacterial proteins identified in amniotic fluid-derived EVs (*n* = 26) and feces-derived EVs (*n* = 25) at the phylum level. The number of protein hits assigned to each phylum is represented in log10-scores, with samples presented in columns and phyla names with ≥ 10 protein hits shown in rows. The sample cohort included a twin pregnancy, designated A and B in the amniotic fluid samples (**A**). GO biological process classes of bacterial proteins identified in AM EVs and FE EVs are presented as log10-scores (**B**). GO molecular function classes of bacterial proteins identified in AM EVs and FE EVs are presented as log10-scores (**C**)

The most identified bacterial phyla in both amniotic fluid and fecal-derived EVs were Bacteroidetes, Firmicutes, Proteobacteria, and Actinobacteria. The gene ontology (GO) annotations were available for 58% (biological process) and 76% (molecular function) of bacterial proteins identified in amniotic fluid EVs, and 9% (biological process) and 12% (molecular function) for feces-derived bacterial EV proteins. The most predominant GOs for biological processes shared by bacterial proteins in amniotic fluid and feces-derived EVs were those for a cellular process [GO:0009987], a metabolic process [GO:0008152], localization [GO:0051179], response to stimulus [GO:0050896], and biological regulation [GO:0065007] (Fig. 2B); for molecular function, the most predominant GOs shared by bacterial proteins in the amniotic fluid and feces-derived EVs were binding [GO:0005488], catalytic activity [GO:0003824], structural molecule activity [GO:0005198], and transporter activity [GO:0005215] (Fig. 2C). Furthermore, translation regulator activity [GO:0045182] was one of the most prevalent GO classes in amniotic fluid EV bacterial proteins but was absent in feces-derived EV bacterial proteins (Fig. 2C).

### EVs in amniotic fluid and maternal feces share a subpopulation of bacterial proteins

**Fig. 3:**
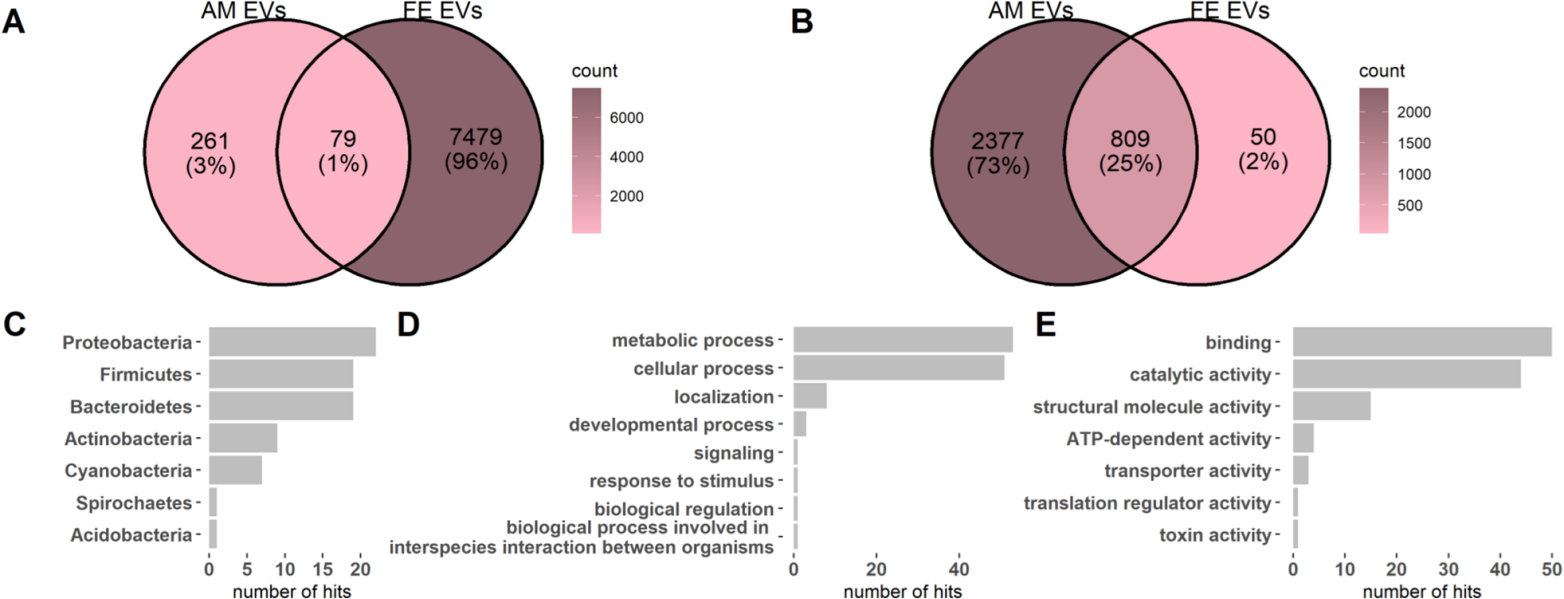
Amniotic fluid-derived extracellular vesicles (AM EVs) and maternal feces-derived extracellular vesicles (FE EVs) share a subpopulation of bacterial proteins. Venn diagram presentation of bacterial protein identifications in AM EVs and FE EVs (**A**). Venn diagram presentation of human protein identifications in AM EVs and FE EVs (**B**). Taxonomy of bacterial proteins identified in both the AM EVs and FE EVs at the phylum level (**C**). GO biological process classes of bacterial proteins identified in both AM EVs and FE EVs (**D**). GO molecular function classes of bacterial proteins identified in both AM EVs and FE EVs (**E**)

Comparing the protein identifications between amniotic fluid-derived and feces-derived EVs, a subpopulation of 79 bacterial proteins (Fig. 3A) and 809 human proteins (Fig. 3B) were identified as present in both types of EV samples. GO annotations for a biological process were available for 63% of the bacterial protein identifications in the overlapping protein set and 68% for molecular function. The predominant GOs for a biological process in the overlapping bacterial protein population were metabolic process [GO:0008152] and a cellular process [GO:0009987] (Fig. 3C) whereas for molecular function, they were binding [GO:0005488] and catalytic activity [GO:0003824] (Fig. 3D). These GO categories are consistent with the overall trends observed in bacterial protein identification in both amniotic fluid EVs and fecal EVs. The overlapping bacterial proteins primarily originated from *Bacteroidetes*, *Firmicutes*, *Proteobacteria*, and *Actinobacteria*, which were the dominant phyla in bacterial protein hits for both amniotic fluid-derived and feces-derived EVs (Fig. 3E). For more detailed information about the overlapping bacterial proteins, please refer to Additional file 1: Table S3.

Among the bacterial proteins identified in the amniotic fluid-derived EVs, two proteins were present in > 80% of the samples originating from the phyla Bacteroidetes and Proteobacteria and two proteins were present in ≥ 50% of the samples originating from Proteobacteria. These proteins are presented in more detail in Additional file 1: Table S4. Among the bacterial proteins identified in the maternal fecal EV samples, 10 proteins mostly originating from the phylum Bacteroidetes were present in > 70% of the samples, whereas six proteins, all originating from the phylum Bacteroidetes, were present in ≥ 60% of the samples. Moreover, 24 bacterial proteins, also mainly originating from Bacteroidetes, were detected in 30–60% of the samples. These proteins are presented in more detail in Additional file 1: Table S5.

### Bacterial 16S rRNA gene analysis suggests a shared origin for bacterial EVs in amniotic fluid and maternal feces

**Fig. 4:**
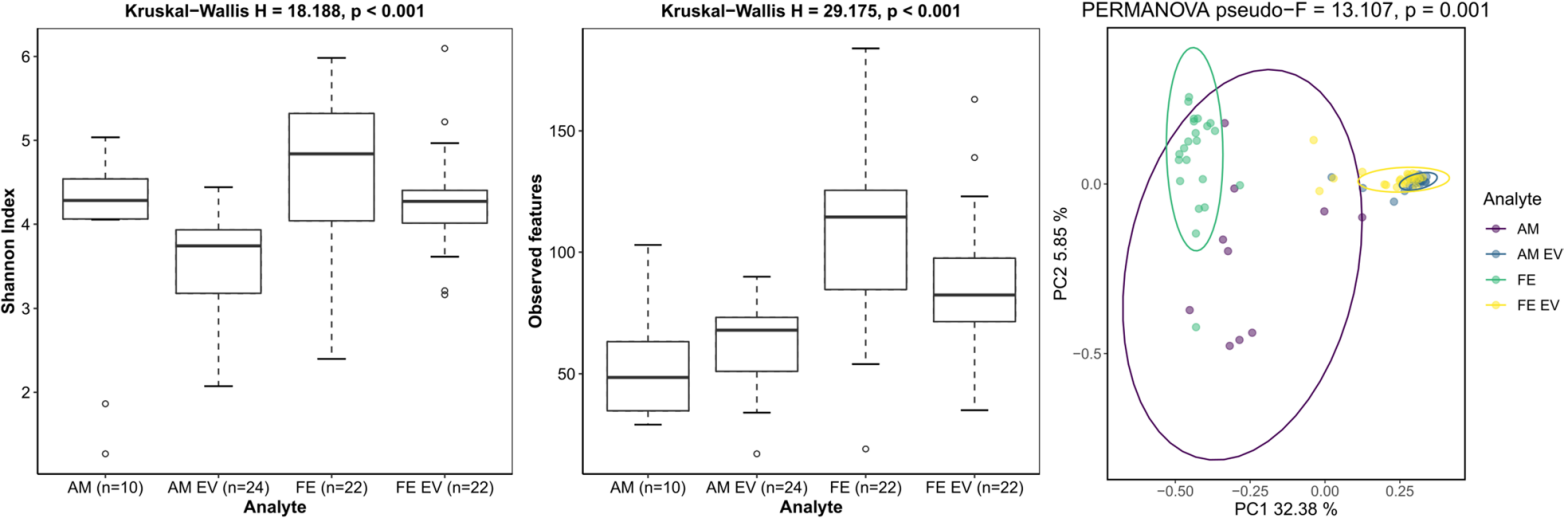
Visualization by principal coordinate analysis (PCoA) shows clustering of the amniotic fluid extracellular vesicles (AM EVs) and maternal fecal extracellular vesicles (FE EVs). Alpha diversity was quantified with the Shannon index and the number of observed features and beta diversity with PCoA using the Bray–Curtis dissimilarity in each sample group (AM = amniotic fluid, AM EVs = amniotic fluid-derived extracellular vesicles, FE = maternal feces, FE EVs = maternal feces-derived extracellular vesicles). The statistical significances of differences between the sample groups were estimated for alpha diversity with the Kruskal–Wallis *H*, as implemented in QIIME2. PERMANOVA was used as the statistical test for differences between the groups, as quantified by the Bray–Curtis dissimilarity. *P* < 0.05 was considered statistically significant

A total of 94 samples, comprising 23 fecal, 23 feces-derived EV, 24 amniotic fluid, and 24 amniotic fluid-derived EV samples, were included in the study. After quality control, 22 fecal, 22 feces-derived EV, 10 AM, and 24 amniotic fluid-derived EV samples were retained for the alpha and beta diversity analyses. Alpha diversity (within-sample diversity) showed significant differences between the sample groups (*p* < 0.05) when calculating the Shannon Index and the number of observed features (Fig. 4). The fecal samples showed higher diversity than the other samples and the maximum variation within the group. Pairwise tests performed using the Wilcoxon rank sum test with Bonferroni correction showed significant differences between the amniotic fluid-derived EV and feces-derived EV, amniotic fluid and amniotic fluid-derived EV, and amniotic fluid-derived EV and fecal groups (Additional file 1: Table S6). Every pairwise comparison of these features yielded significant differences, except for those between the amniotic fluid-and amniotic fluid-derived EV groups and the fecal and feces-derived EV groups. All sample groups showed significant differences in beta diversity (between-sample diversity). Visualization by principal coordinate analysis with Bray–Curtis dissimilarity suggested clustering of the samples according to their predefined groupings and clustering of the amniotic fluid EVs and maternal fecal EVs (Fig. 4). Group-wise and pairwise comparisons with PERMANOVA confirmed that the differences in the composition between the sample groups were significant (*p* = 0.001) (Fig. 4, Additional file 1: Table S6).

**Fig. 5:**
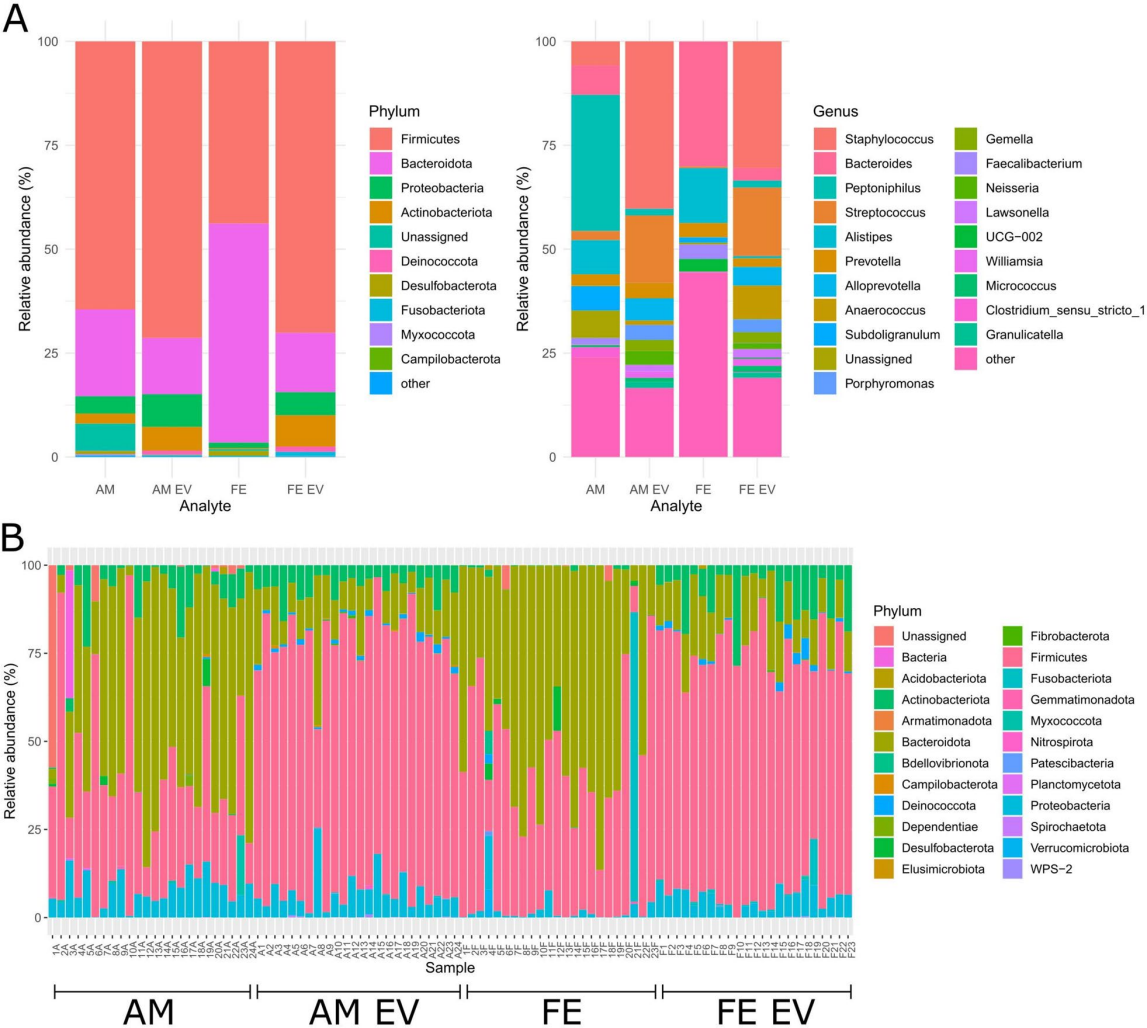
Amniotic fluid extracellular vesicles (AM EVs) and maternal fecal extracellular vesicles (FE EVs) show similarities in their bacterial compositions. Taxa bar plots at the phylum and genus levels based on the sample type (AM = amniotic fluid, AM EVs = amniotic fluid-derived extracellular vesicles, FE = maternal feces, FE EVs = maternal feces-derived extracellular vesicles). At the phylum level, the 10 most frequent phyla are colored in, and the rest are collapsed to form the “other” category, whereas at the genus level, the 20 most frequent genera are colored in and the rest collapsed to an “other” category (**A**). Relative abundances of all phyla in each sample (**B**)

The taxonomic analyses of the sample groups showed a variation in the abundance of bacteria. Although approximately all samples had bacterial 16S rRNA gene findings indicative of the phyla *Firmicutes* and *Bacteroidota* (*Bacteroides* in previous 16S rRNA gene sequence analysis databases) (Fig. 5), the fecal samples mostly comprised *Bacteroides* (30%) and *Alistipes* (13%) at the genus level, whereas the most common genus in the amniotic fluid samples was *Peptoniphilus* (33%), with the other genera accounting for < 10% of the bacterial composition (Additional file 1: Table S7). Conversely, the EV samples were similar in their bacterial compositions, with slightly varying abundances of mostly *Staphylococcus* and *Streptococcus* (Additional file 1: Table S7).

The differential abundance analysis with ANCOM between the sample groups revealed many differentially abundant taxa at the phylum and genus levels: the FE versus FE EV samples had 36, the AM versus AM EV samples had 22, the FE versus AM samples had 6, and the FE EV versus AM EV samples had 4 such taxa (Additional file 1: Supplementary Figure S1, Supplementary Table S8).

### Human maternal fecal EVs reach the fetus in pregnant mice

**Fig. 6:**
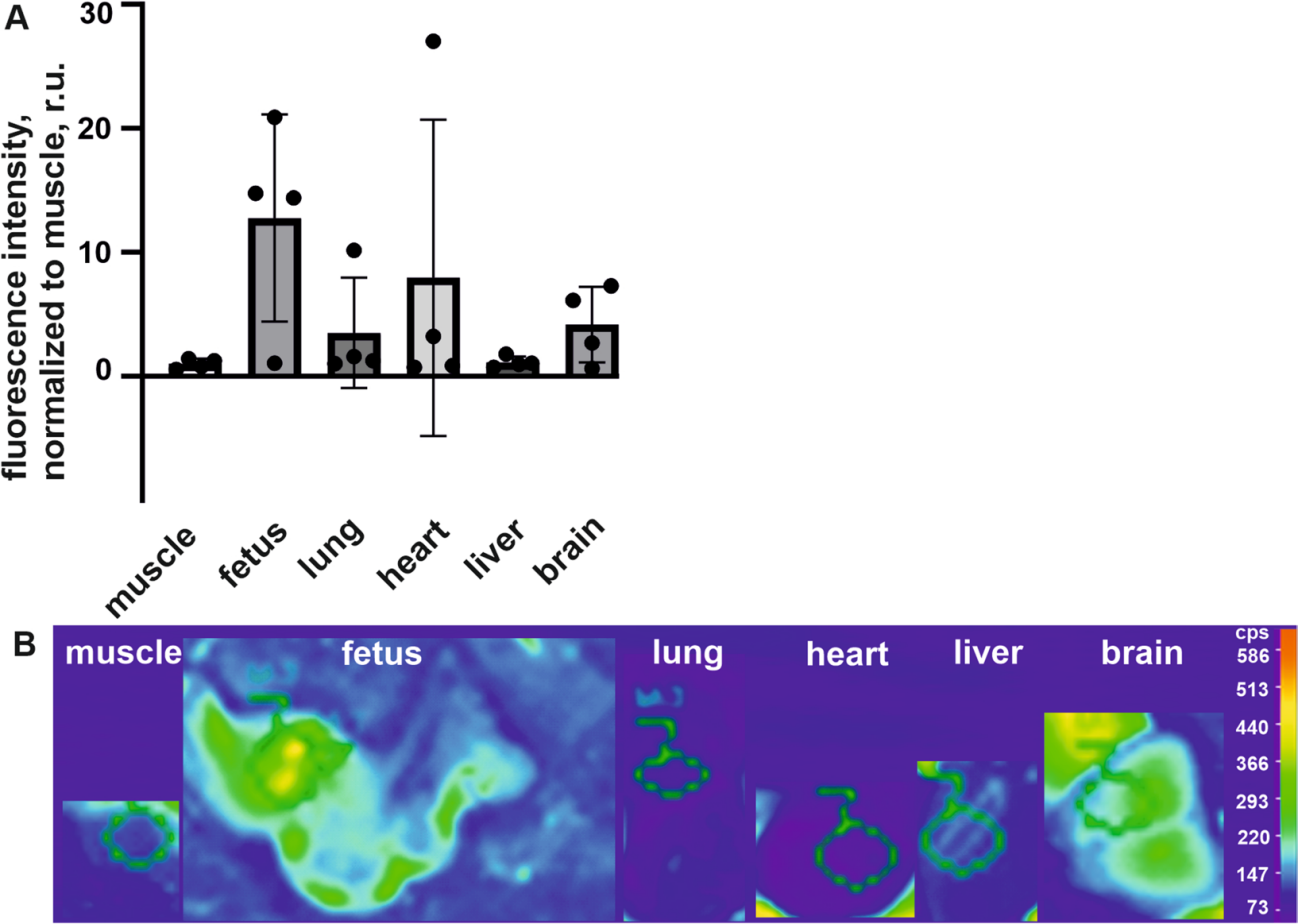
Fecal extracellular vesicles (EVs) of pregnant women show accumulation to fetus in pregnant mice. Labeled EVs isolated from the feces of pregnant women were injected intravenously into the mice at the end of the first stage of pregnancy. The animals were sacrificed 24 h after injection, and images of various organs and fetuses were obtained. Fluorescent intensity was assessed in various maternal organs and fetuses. The data are shown as means, with standard deviation shown by the error bar, *n* = 4 mice, nonsignificant (ns) between organs/fetuses in the Kruskal–Wallis nonparametric test (**A**). Representative fluorescence image of the organs and fetuses indicated, with regions of interest (ROI) of similar size are drawn in (**B**)

The translocation of maternal gut microbiota EVs to the fetus was tested in our mouse model through EVs derived from fecal samples of pregnant women. The fluorescence-labeled EVs were injected into the tail vein of the pregnant mice, and the results showed specific accumulation in mouse fetuses 24 h after injection. Additionally, these labeled EVs were found to accumulate in distal organs, including the maternal lung, heart, liver, and brain (Fig. 6).

## Discussion

The findings of the present study demonstrated that microbiota-derived EVs are present in the amniotic fluid of pregnant women. Notably, EVs found in the amniotic fluid and maternal feces exhibit similarities in terms of their protein cargo and bacterial composition. In a mouse model, we successfully isolated EVs from human maternal fecal samples, which were then shown to reach the intra-amniotic space. We found that EVs originating from the maternal microbiota can reach the fetal environment, which is a previously unreported interaction mechanism between the maternal gut microbiota and the developing fetus. The presence of maternal gut microbiota-derived EVs in the amniotic fluid suggests a plausible mechanism for priming the fetal immune system, which is crucial for neonatal gut colonization after birth.

Previous research on bacterial EVs in fetal environments has often focused on studying the effects of EVs isolated from single bacterial pathogenic strains in animal models investigating pregnancy complications [20, 21]. Furthermore, investigations into the presence of bacteria in the fetal environment have mainly relied on 16S rRNA gene sequencing, with limited emphasis on other biomolecules of bacterial origin [47]. A recent study by Nunzi et al. (2023) explored the monocyte activation potential of amniotic fluid-derived EVs, and reported the presence of bacterial EVs in human amniotic fluid via 16S-rRNA gene sequencing [24]. Similarly, Menon et al. (2023) examined the placental microbiome through 16S rRNA gene sequencing of bacterial EVs from the placenta [25]. In contrast to these previous publications, our study takes a more comprehensive approach by characterizing amniotic fluid-derived bacterial EVs through proteomics in addition to 16S rRNA gene analysis, facilitating a direct comparison with maternal gut microbiota EVs.

The present study showed the presence of bacterial EVs in human amniotic fluid, as evidenced by bacterial proteins and RNA. Proteomic analysis in the present study revealed a subgroup of bacterial proteins existing in both amniotic fluid-derived and maternal feces-derived EVs. The protein cargo in both EV groups was observed to originate mostly from the same bacterial phyla, exhibiting similar functional characteristics. Despite differences observed in the bacterial DNA of amniotic fluid and maternal fecal samples in the 16S rRNA gene analysis, the EVs isolated from these sources demonstrated similarities in bacterial composition and diversity, suggesting a common source. These results support the hypothesis that maternal microbiota communicates with the developing fetus during pregnancy through microbiota-derived EVs.

Our mouse model experiments provide further evidence that maternal microbiota-derived EVs can reach the fetus during pregnancy. Previous research has indicated that bacterial EVs possess the capability to cross the intestinal epithelial barrier, enter the bloodstream, and subsequently translocate to distal organs. This phenomenon has previously been described either in association with occurrences of gut-associated bacterial EVs in human body fluids, such as blood [48] and breastmilk [49] or through biodistribution assays using labeled bacterial EVs in animal models that were performed outside the scope of pregnancy and with bacterial derived from cultured strains of commensal bacteria [50, 51]. In the present study, bacterial EVs were isolated from fecal samples, thus representing the entire EV secretion from the maternal gut microbiota in order to assess its biodistribution to the fetus.

The first-pass meconium of the infant formed in utero already harbors a distinctive microbiota [13, 52–54]; however, the fetal microbiome concept of the amniotic fluid and placenta has provoked criticism because distinctive fetal microbiota comprising whole-cell bacteria appears to be unlikely [13–19]. However, a study by Jimenez et al. (2008) reported that the DNA of *Enterococcus faecium* was detectable in the amniotic fluid and meconium when bacteria were orally inoculated into pregnant mice [54]. The translocation of maternal microbiota-derived EVs may help reconcile these conflicting findings, providing a potential explanation for the presence of bacterial material in fetal environments without the need for live bacteria. Hence, future investigations into the concept of a fetal microbiome should consider the role of maternal microbiota-derived EVs.

The development of the human fetal immune system begins early in gestation [55]. Mishra et al. (2021) suggested that microbial exposure during gestation primes fetal immune cells [56], as they detected the presence of bacteria in multiple fetal tissues and examined the activation of fetal memory T cells in response to microbial exposure. In addition to commensal bacteria, microbial metabolites have been shown to regulate intestinal adaptive immune responses, including the modulation of regulatory T cells [57]. Bacterial EVs can activate various host immune responses through pathogen-associated molecular patterns and the subsequent activation of toll-like receptor 2 and toll-like receptor 4 [58–62]. When the fetus ingests amniotic fluid in utero, bacterial EVs from the amniotic fluid likely reach the gut epithelia, providing a safe pathway for early bacterial exposure through these non-replicating bacterial units. Based on the results of the present study, we hypothesize that maternal gut microbiota-derived EVs, present in amniotic fluid and likely ingested by the fetus, guide the fetal immune system toward the immune tolerance required for early gut colonization at birth. Our findings support the idea that bacterial EVs from the maternal gut microbiota are a natural part of the fetal environment during a healthy pregnancy.

The main strength of our study lies in its comprehensive examination of maternal gut microbiota and amniotic fluid EVs. Additionally, we assessed bacterial EVs in the amniotic fluid in terms of both protein and RNA. The analysis of two distinct groups of biomolecules enables a more thorough understanding of EV secretion and their cargo. However, some limitations of our study should be considered. Separating EVs of host and microbiota origin remains challenging, and our mouse biodistribution assay might have represented a mixture of host and microbiota EVs, despite our isolation protocol involving bacterial EV enrichment. Additionally, the low biomass of samples in 16S rRNA gene amplicon analysis poses a risk of contamination, which we addressed by implementing the guidelines for good practices and controls described by de Goffau et al*.* (2018) [63]. Finally, although our focus was on exploring gut microbiota-derived EVs in the fetal environment, the potential contribution of bacterial EVs from other sources, such as the oral or vaginal microbiota, warrants further investigation.

## Conclusions

Our findings demonstrate that bacterial EVs secreted by the maternal gut microbiota reach the intra-uterine space during fetal development. This discovery suggests a mechanism for interaction between the maternal microbiota and the fetus, which may play a crucial role in preparing the fetal immune system for early gut colonization at birth.

### Supplementary Information

**Additional file 1.** Supplementary tables and figures.

**Additional file 2.** Kaisanlahti_AMBI_proteomics.

**Additional file 3.** Proteomics metadata.

**Additional file 4.** README_proteomics.

**Additional file 5.** Proteins_swissprot.

**Additional file 6.** Proteins_trEMBL.

## Data Availability

The 16S rRNA gene sequencing datasets generated and analyzed during the current study are available in the Genbank repository with bioproject accession: PRJNA878641. The mass spectrometry proteomics data have been deposited to the ProteomeXchange Consortium via the PRIDE [64] partner repository with the dataset identifier PXD045755.

## Abbreviations

EVs: Extracellular vesicles
PBS: Phosphate-buffered saline
PCR: Polymerase chain reaction
ANCOM: Analysis of composition of microbiomes
GO: Gene ontology
