# Supplementary material for: Maternal microbiota communicates with the fetus through microbiota-derived extracellular vesicles
Source: Microbiome. 2023 Nov 13;11:249. doi: 10.1186/s40168-023-01694-9 (PMC10642029; doi:10.1186/s40168-023-01694-9)
Supplement: Supplementary file 2 — Additional file 1. Supplementary tables and figures. [file 40168_2023_1694_MOESM1_ESM.pdf]

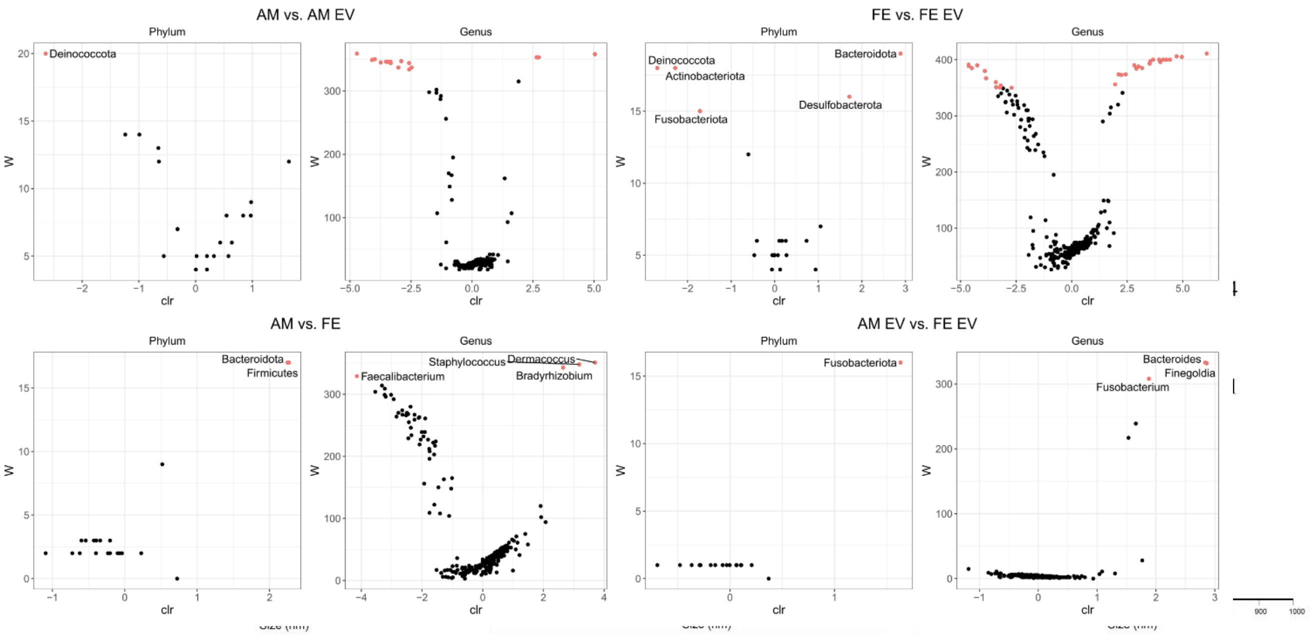

Fig. 1. ANCOM analysis of the differentially abundant taxa in the sample groups. In the analysis, the centered log ratio (clr) represents the effect size and W the number of times the 0-hypothesis has been rejected. Differentially abundant taxa are identified at the phylum and genus levels and marked as red dots in the volcano plots. The differentially abundant genera in the maternal feces (FE) vs. maternal feces-derived extracellular vesicles (FE EV) and amniotic fluid (AM) vs. amniotic fluid-derived extracellular vesicles (AM EV) comparisons are listed in Supplementary Table 8.

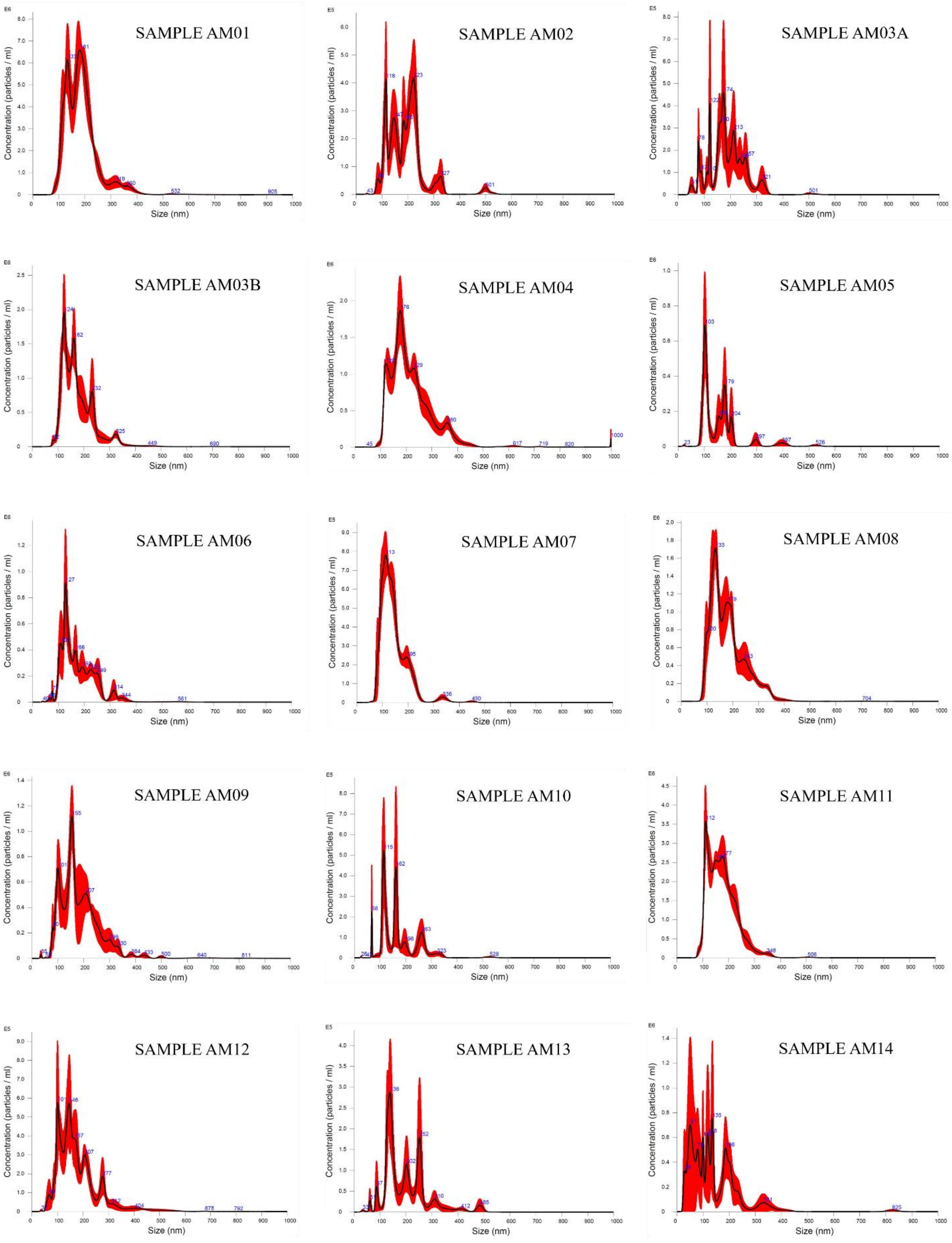
SAMPLE AM01
SAMPLE AM02
SAMPLE AM03A
SAMPLE AM03B
SAMPLE AM04
SAMPLE AM05
SAMPLE AM06
SAMPLE AM07
SAMPLE AM08
SAMPLE AM09
SAMPLE AM10
SAMPLE AM11
SAMPLE AM12
SAMPLE AM13
SAMPLE AM14
Concentration (particles / ml)
Size (nm)

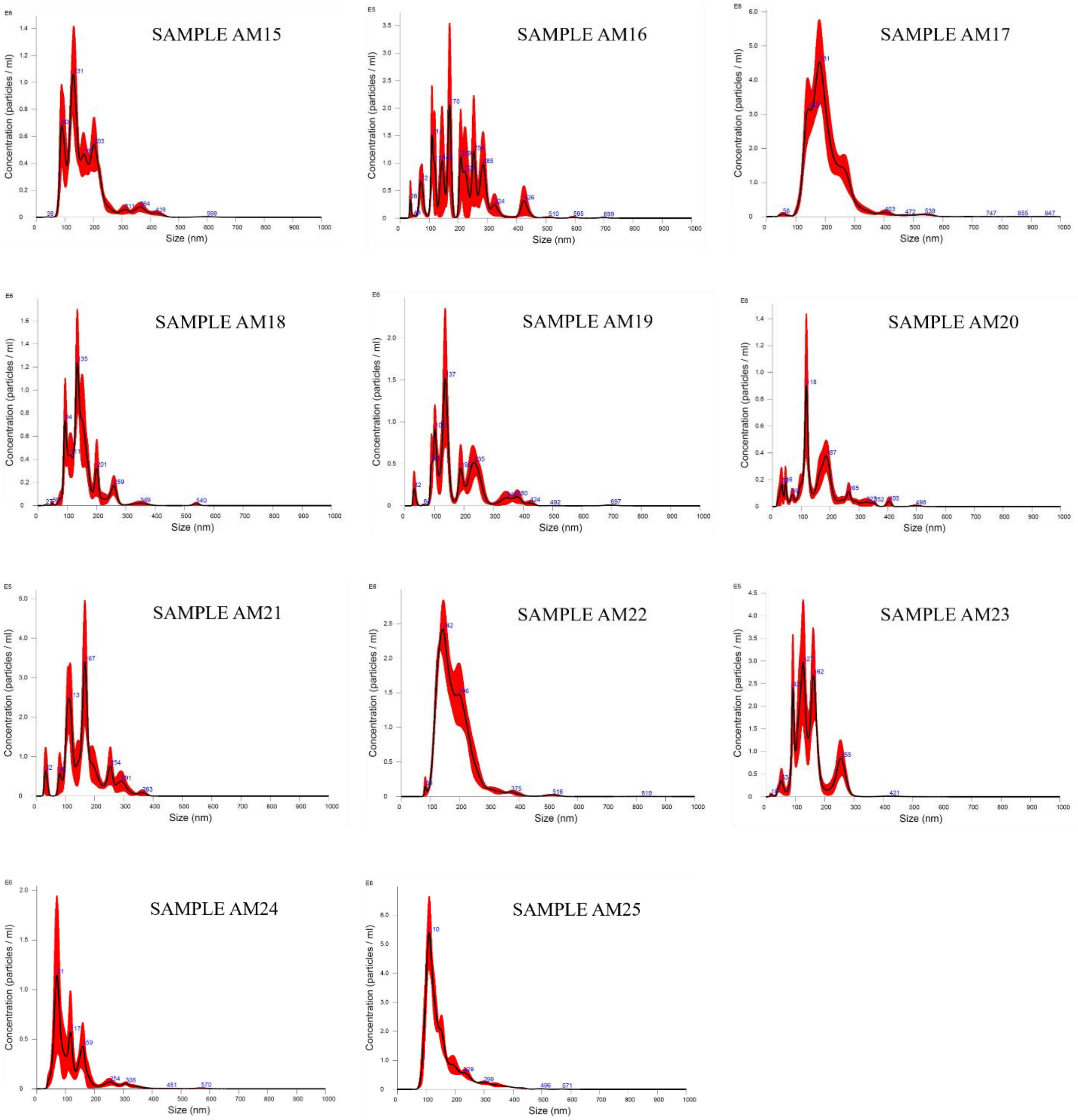

Fig. 2. Size distribution analysis of amniotic fluid-derived extracellular vesicles (EVs) by nanoparticle tracking analysis. Samples AM01 – AM25.

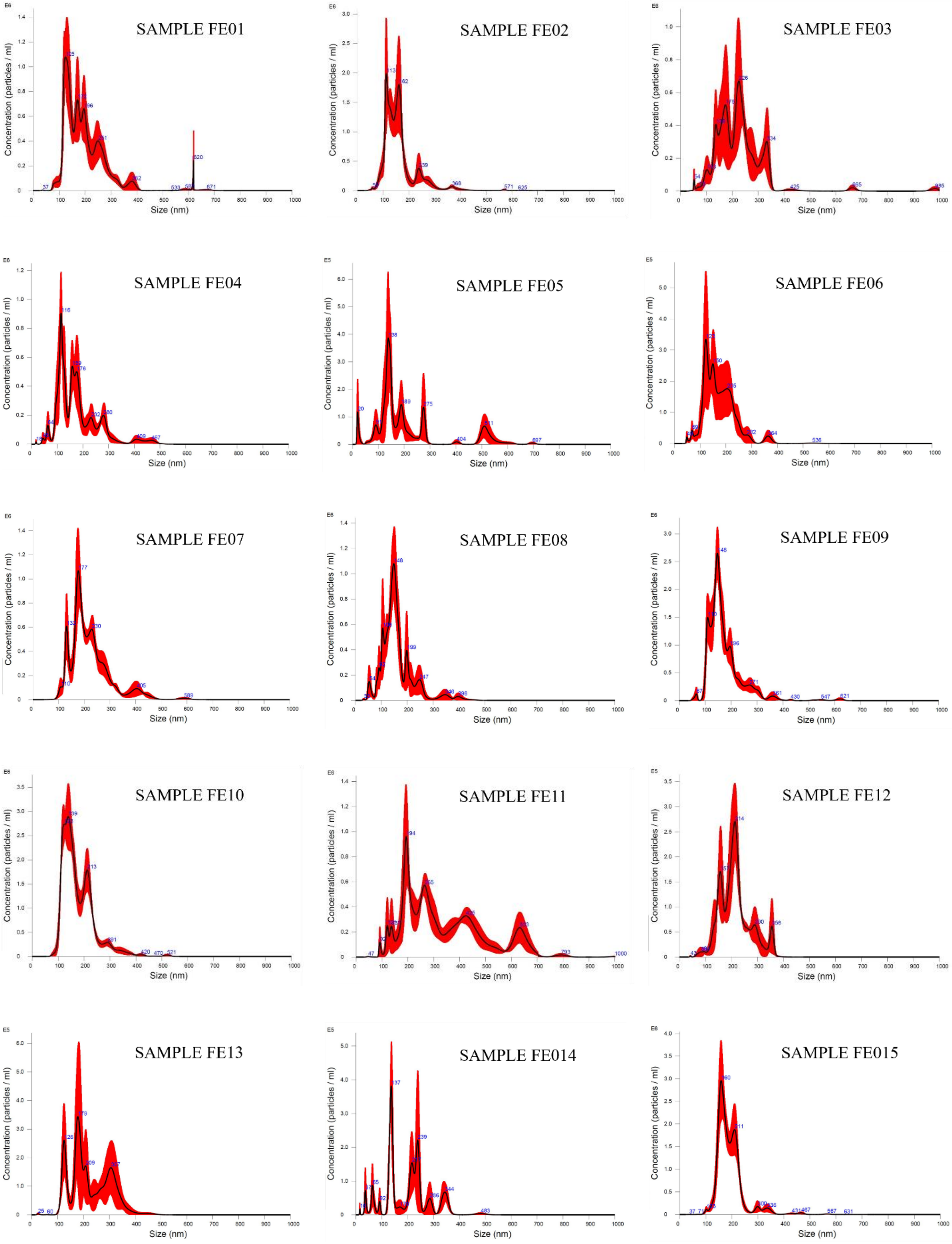
SAMPLE FE01
SAMPLE FE02
SAMPLE FE03
SAMPLE FE04
SAMPLE FE05
SAMPLE FE06
SAMPLE FE07
SAMPLE FE08
SAMPLE FE09
SAMPLE FE10
SAMPLE FE11
SAMPLE FE12
SAMPLE FE13
SAMPLE FE014
SAMPLE FE015
Concentration (particles / ml)
Size (nm)

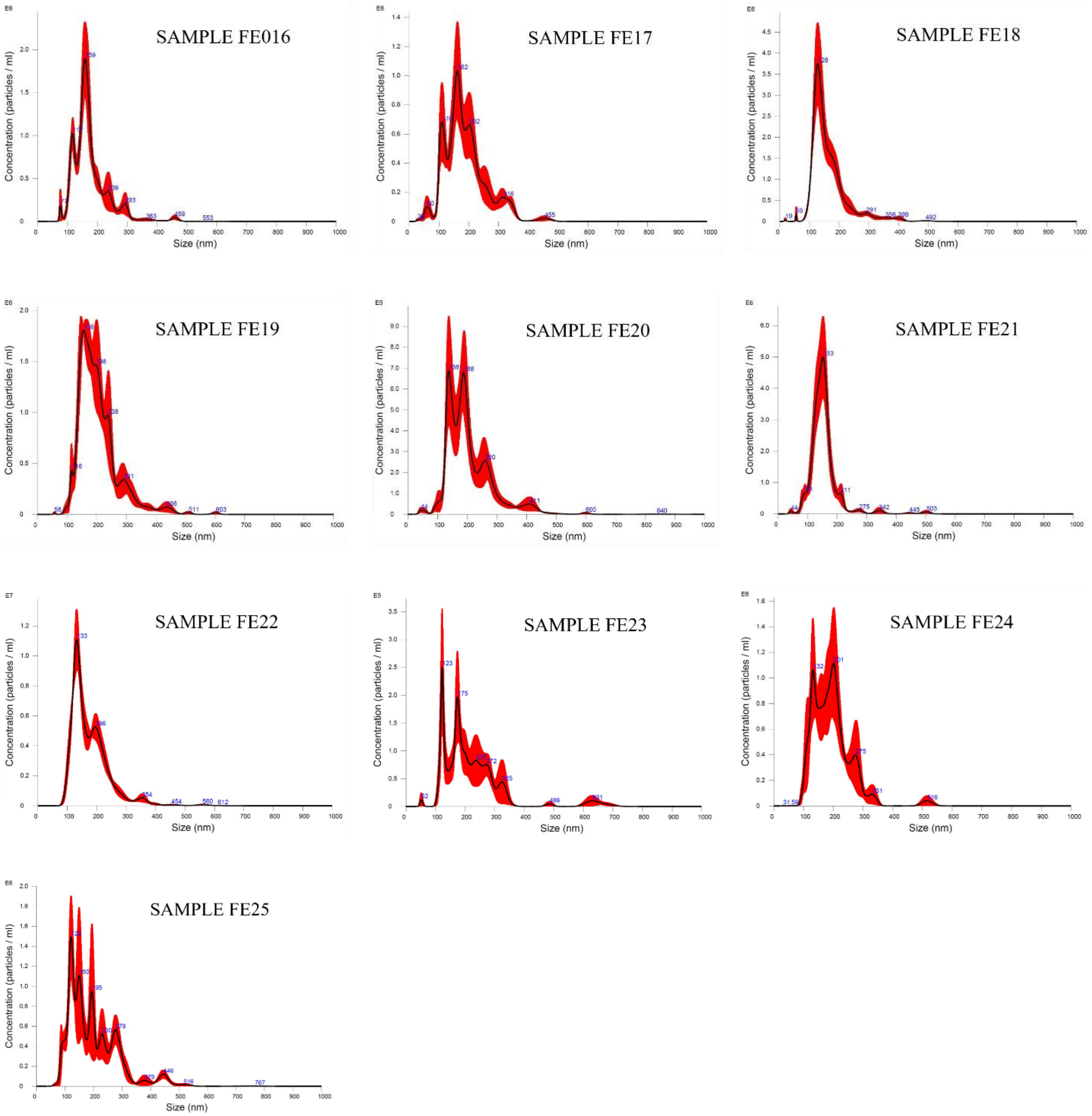

Fig. 3. Size distribution analysis of maternal feces-derived extracellular vesicles (EVs) by nanoparticle tracking analysis. Samples FE01 – FE25.

**Population characteristics**	
Maternal characteristics	**N=28[a]**
Maternal age (years) Mean (SD)	31 (4.8)
Number of siblings Mean (SD)	0.9 (0.9)
Maternal asthma N (%)	2 (7.1)
Maternal allergy N (%)	9 (32)
Gestational diabetes N (%)	11 (39)
Smoking during pregnancy	6 (21)
S. agalactiae screen positive [b]	5 (18)
Antibiotics during pregnancy [c]	4 (14)
Antibiotics during delivery [d]	28 (100)
Newborn characteristics	
Female (%)	13 (46)
Gestational age (weeks) Mean (SD)	38.5 (1.5)
Birth weight (grams) Mean (SD)	3420 (580)
Apgar 1 min Mean (SD)	8.4 (1.3)
Apgar 5 min Mean (SD)	8.8 (1.1)
Apgar 15 min Mean (SD)	9.0 (0.7)
Antibiotics after birth N (%)[f]	3 (11)

[a] Both fecal and amniotic fluid samples were available for 22 cases, fecal samples only for 3 cases and amniotic fluid samples only for 3 cases.
[b] In 9 cases *Str. agalactiae* screening was not performed.
[c] One mother had received pivmecillinam and one metronidazole. In two cases the antibiotic used was not recorded.
[d] All except one received cefuroxime
[f] Three infants received benzylpenicillin and tobramycin.

Table 1. Amniotic fluid and fecal samples: clinical data on the pregnant women.

	AMNIOTIC FLUID	FECES
Number of protein identifications	3526	8417
Bacterial proteins	340	7558
Mean number of bacterial proteins per sample (range)	30 (2-190)	1333 (10-3671)
Human proteins	3186	859
Mean number of human proteins per sample (range)	601 (71-2217)	60 (4-707)

Table 2. Proteins identified in the amniotic-fluid and feces-derived EVs.

PROTEIN NAME	PHYLUM	HITS
High-molecular weight cobalt-containing nitrile hydratase subunit alpha, H-Nhase, H-nitrilase, EC 4.2.1.84	Actinobacteria	1
Flagellin	Firmicutes	4
Uncharacterized protein	Proteobacteria, Actinobacteria,,	6
tRNA (guanine-N(7)-)-methyltransferase	Proteobacteria	1
Enolase, EC 4.2.1.11	Spirochaetes, Proteobacteria,	3
30S ribosomal protein S8	Proteobacteria	1
AAA-family ATPase	Actinobacteria	2
Tubulin_C domain-containing protein	Actinobacteria	1
Translation elongation factor EF-1 subunit alpha	Actinobacteria	1
30S ribosomal protein S13	Proteobacteria	1
30S ribosomal protein S19	Proteobacteria, Actinobacteria	3
Putative membrane protein YgcG	Actinobacteria	1
Pesticidal crystal protein Cry2Ab	Firmicutes	1
Fumarate reductase	Bacteroidetes	2
TonB-dependent receptor	Bacteroidetes	1
ATP-dependent zinc metalloprotease FtsH,	Actinobacteria	1
2-isopropylmalate synthase, EC 2.3.3.13	Firmicutes	2
Subtilisin BL, EC 3.4.21.62	Firmicutes	2
M protease, EC 3.4.21.-	Firmicutes	1
Alkaline protease, EC 3.4.21.-	Firmicutes	2
Ribulose bisphosphate carboxylase large chain, RuBisCO large	Cyanobacteria	6
OmpA-family protein	Proteobacteria, Bacteroidetes	12
DNA, contig: SP617	Proteobacteria	1
Outer membrane protein OmpA-like peptidoglycan-associated	Proteobacteria	1
TIGR01841-family phasin	Proteobacteria	1
Phasin-family protein	Proteobacteria	1
DNA, contig: SP655	Proteobacteria	1
TonB-linked outer membrane protein, SusC/RagA family	Proteobacteria, Bacteroidetes	2
MotA/TolQ/ExbB proton channel-family protein	Proteobacteria	5
Glutamate synthase [NADPH] large chain, EC 1.4.1.13	proteobacteria	1
Glyceraldehyde-3-phosphate dehydrogenase, GAPDH, EC	Firmicutes	6
Porin	Bacteroidetes	2
DUF4988 domain-containing protein	Bacteroidetes	1

Table 3. Bacterial protein identifications in the proteomic analyses of the amniotic fluid extracellular vesicles and maternal fecal extracellular vesicles.

	PROTEIN NAME	PHYLUM	HITS
>80%	Contig40, whole genome shotgun sequence	Bacteroidetes	1
	IPTL-CTERM sorting domain-containing protein (Fragment)	Proteobacteria	1
≥50%	tRNA (guanine-N(7)-)-methyltransferase	Proteobacteria	1
	Uncharacterized protein	Proteobacteria	2
>25%	Chaperone protein DnaK (HSP70) (Heat shock protein 70)	Thermotogae<br>Chlorobi<br>Chlorobi<br>Cyanobacteria	4
	Glyceraldehyde-3-phosphate dehydrogenase 1 (GAPDH 1)	Firmicutes	7
	Uncharacterized protein (Fragment)	Actinobacteria<br>Proteobacteria	3
	RRM domain-containing protein	Armatimonadetes	1
	Ubiquitin-like domain-containing protein	Proteobacteria	1
	AAA-family ATPase (Fragment)	Actinobacteria	1

Table 4. Distribution of bacterial protein identifications in amniotic fluid EV samples.

	PROTEIN NAME	PHYLUM	HITS
>70%	TonB-dependent receptor	Bacteroidetes	17
	SusC/RagA-family TonB-linked outer membrane protein	Bacteroidetes	27
	Uncharacterized protein	Bacteroidetes	5
	TonB-dependent receptor plug domain-containing protein	Bacteroidetes	3
	SusC protein	Bacteroidetes	2
	Flagellin	Firmicutes	2
	Tetratricopeptide repeat protein	Bacteroidetes	19
	Vitamin B12 transporter BtuB	Bacteroidetes	1
	Iron complex outer membrane receptor protein	Bacxteroidetes	1
	TPR domain protein	Bacteroidetes	1
≥60%	(Putative) Outer membrane protein	Bacteroidetes	11
	SusD/RagB-family nutrient-binding outer membrane lipoprotein	Bacteroidetes	97
	Outer membrane cobalamin receptor protein	Bacteroidetes	4
	Carboxypeptidase regulatory-like domain protein	Bacteroidetes	3
	Membrane protein	Bacteroidetes	2
	Uncharacterized protein	Bacteroidetes	29
>50%	High-molecular weight cobalt-containing nitrile hydratase subunit alpha (H-Nhase) (H-nitrilase) (EC 4.2.1.84)	Actinobacteria	1
	OmpA-family protein	Bacteroidetes	25
	Porin-family protein/Outer membrane beta-barrel domain protein	Bacteroidetes	1
	Cell surface protein	Bacteroidetes	15
	Conserved hypothetical exported protein	Bacteroidetes	1
	Hemin receptor	Bacteroidetes	4
	Uncharacterized protein	Bacteroideted	85
≥30%	Formate-tetrahydrofolate ligase (EC 6.3.4.3)	Firmicutes	18
	Ferrienterobactin receptor	Bacteroidetes	3
	OMP_b-brl domain-containing protein	Bacteroidetes	18
	NAD-specific glutamate dehydrogenase (NAD-GDH)	Bacteroidetes	1
	DNA, contig: SP617	Proteobacteria	1
	STN domain-containing protein	Bacteroidetes	2
	Mucoidy inhibitor MuiA family protein	Bacteroidetes	1
	Ferritin (EC 1.16.3.2)	Bacteroidetes	23
	Major outer membrane lipoprotein Lpp	Proteobacteria	32
	Adenine deaminase (ADE) (EC 3.5.4.2)	Proteobacteria	1
	SLH domain-containing protein	Firmicutes	2
	Putative cell surface protein	Bacteroidetes	3
	MotA/TolQ/ExbB proton channel-family protein	Bacteroidetes	13
	DUF3869 domain-containing protein	Bacteroidetes,	21
	Collagen triple helix repeat (20 copies)	Bacteroidetes	1
	DUF4988 domain-containing protein	Bacteroidetes	2

Table 5. Distribution of bacterial protein identifications in the maternal fecal EV samples.

		Shannon Index	Observed features	Bray-Curtis dissimilarity
Group 1	Group 2	p-value	p-value	p-value
AM (n=10)	AM_EV (n=24)	0.186	0.576	**0.001**
AM (n=10)	FE (n=22)	0.713	**0.002**	**0.001**
AM (n=10)	FE_EV (n=22)	1.000	**0.025**	**0.001**
AM_EV (n=24)	FE (n=22)	**0.003**	**< 0.001**	**0.001**
AM_EV (n=24)	FE_EV (n=22)	**0.001**	**0.010**	**0.001**
FE (n=22)	FE_EV (n=22)	1.000	0.150	**0.001**

Table 6. Pairwise results of the Wilcoxon rank sum test for Shannon Index, Observed features and PERMANOVA for Bray-Curtis dissimilarity. The p-values for the Shannon Index and Observed features are adjusted using the Bonferroni correction.

Maternal feces			
Phylum (%)		Genus (%)	
Bacteroidota	53	*Bacteroides*	30
Firmicutes	44	*Alistipes*	13
Proteobacteria	1.3	*Faecalibacterium*	3.5
Desulfobacterota	1.2	*Prevotella*	3.4
Unassigned	0.44	UCG-002	3.1
Actinobacteriota	0.30	*Turicibacter*	2.4
Fusobacteriota	0.24	Erysipelotrichaceae_UCG-003	2.1
Campilobacterota	0.020	*Barnesiella*	2.1
Verrucomicrobiota	0.014	Clostridia_UCG-014	1.9
Spirochaetota	0.0070	*Blautia*	1.8
other	0.0088	other	37
**Amniotic fluid**			
**Phylum (%)**		**Genus (%)**	
Firmicutes	65	*Peptoniphilus*	33
Bacteroidota	21	*Alistipes*	8.2
Unassigned	6.6	*Bacteroides*	7.1
Proteobacteria	4.2	*Unassigned*	6.6
Actinobacteriota	2.4	*Subdoligranulum*	5.9
Desulfobacterota	0.68	*Staphylococcus*	5.8
Myxococcota	0.28	*Prevotella*	2.7
Dependentiae	0.15	*Clostridium_sensu_stricto_1*	2.4
d__Bacteria	0.14	*Streptococcus*	2.2
Campilobacterota	0.069	f__Oscillospiraceae_NK4A214_group	1.6
other	0.14	Other	25
**Feces-derived extracellular vesicles**			
**Phylum (%)**		**Genus (%)**	
Firmicutes	70	*Staphylococcus*	31
Bacteroidota	14	*Streptococcus*	17
Actinobacteriota	7.5	*Anaerococcus*	8.1
Proteobacteria	5.6	*Alloprevotella*	4.5
Deinococcota	1.3	*Bacteroides*	3.0
Fusobacteriota	0.97	*Porphyromonas*	3.0
Fibrobacterota	0.064	*Gemella*	2.6
WPS-2	0.057	*Prevotella*	2.1
Acidobacteriota	0.046	*Lawsonella*	2.1
Myxococcota	0.033	*Williamsia*	1.7
other	0.051	other	26
**Amniotic fluid-derived extracellular vesicles**			
**Phylum (%)**		**Genus (%)**	
Firmicutes	71	*Staphylococcus*	40
Bacteroidota	14	*Streptococcus*	16
Proteobacteria	7.9	*Alloprevotella*	5.3
Actinobacteriota	5.7	*Prevotella*	3.7
Deinococcota	0.97	*Porphyromonas*	3.6
Campilobacterota	0.15	*Neisseria*	3.3
Fibrobacterota	0.12	*Gemella*	2.7
WPS-2	0.11	*Lawsonella*	1.6
Fusobacteriota	0.066	*Peptoniphilus*	1.6
Nitrospirota	0.062	*Dolosigranulum*	1.5
other	0.054	other	20

Table 7. Relative abundance of the ten most abundant phyla and genera in each group of samples.

FE vs. FE EV				AM vs. AM EV			
Phylum	w	Genus	w	Phylum	w	Genus	w
Bacteroidota	19	*Staphylococcus*	411	Deinococcota	20	*Alloprevotella*	359
Actinobacteriota	18	*Porphyromonas*	406			*Bacteroides*	358
Deinococcota	18	*Alloprevotella*	405			*Alistipes*	358
Desulfobacterota	16	*Granulicatella*	400			*Dermacoccus*	353
Fusobacteriota	15	*Neisseria*	400			*Bradyrhizobium*	353
		Gemella	400			*Vibrionimonas*	353
		Peptoniphilus	400			*Gemella*	350
		Lawsonella	400			*Porphyromonas*	349
		Finegoldia	397			*Deinococcus*	347
		Anaerococcus	396			*Granulicatella*	346
		Micrococcus	393			*Dolosigranulum*	346
		Faecalibacterium	391			*Neisseria*	346
		Dolosigranulum	390			*Lawsonella*	346
		Eubacterium_coprostanoligenes_group	390			*Williamsia*	346
		Alistipes	389			*Streptococcus*	345
		Deinococcus	388			*Staphylococcus*	344
		Williamsia	385			*Veillonella*	344
		Bacteroides	385			*Escherichia-Shigella*	337
		Streptococcus	384			*Peptoniphilus*	337
		Oscillibacter	380			*Prevotella*	334
		Veillonella	374				
		Ezakiella	374				
		Eremococcus	373				
		Barnesiella	367				
		Blautia	360				
		Parvimonas	356				
		Christensenellaceae_R-7_group	354				
		f__Ruminococcaceae_uncultured	353				
		Subdoligranulum	351				
		f__Lachnospiraceae	350				
		Colidextribacter	350				
FE vs. AM				FE EV vs. AM EV			
phylum	w	genus	w	phylum	w	genus	w
Bacteroidota	17	*Dermacoccus*	351	Fusobacteriota	16	*Bacteroides*	333
Firmicutes	17	*Staphylococcus*	348			*Finegoldia*	332
		Bradyrhizobium	343			*Fusobacterium*	308
		Faecalibacterium	329				

Table 8. ANCOM analysis of differentially abundant taxa in the sample groups at the phylum and genus levels.
